# A Glycoprotein Mutation That Emerged during the 2013–2016 Ebola Virus Epidemic Alters Proteolysis and Accelerates Membrane Fusion

**DOI:** 10.1128/mBio.03616-20

**Published:** 2021-02-16

**Authors:** J. Maximilian Fels, Robert H. Bortz, Tanwee Alkutkar, Eva Mittler, Rohit K. Jangra, Jennifer S. Spence, Kartik Chandran

**Affiliations:** a Department of Microbiology and Immunology, Albert Einstein College of Medicine, Bronx, New York, USA; Duke University Medical Center

**Keywords:** Ebola virus, proteolysis, variant surface glycoprotein

## Abstract

Genomic surveillance of viral isolates during the 2013–2016 Ebola virus epidemic in Western Africa, the largest and most devastating filovirus outbreak on record, revealed several novel mutations. The responsible strain, named Makona, carries an A-to-V substitution at position 82 (A82V) in the glycoprotein (GP), which is associated with enhanced infectivity *in vitro*. Here, we investigated the mechanistic basis for this enhancement as well as the interplay between A82V and a T-to-I substitution at residue 544 of GP, which also modulates infectivity in cell culture. We found that both 82V and 544I destabilize GP, with the residue at position 544 impacting overall stability, while 82V specifically destabilizes proteolytically cleaved GP. Both residues also promote faster kinetics of lipid mixing of the viral and host membranes in live cells, individually and in tandem, which correlates with faster times to fusion following colocalization with the viral receptor Niemann-Pick C1 (NPC1). Furthermore, GPs bearing 82V are more sensitive to proteolysis by cathepsin L (CatL), a key host factor for viral entry. Intriguingly, CatL processed 82V variant GPs to a novel product with a molecular weight of approximately 12,000 (12K), which we hypothesize corresponds to a form of GP that is pre-triggered for fusion. We thus propose a model in which 82V promotes more efficient GP processing by CatL, leading to faster viral fusion kinetics and higher levels of infectivity.

## INTRODUCTION

Ebola virus (EBOV), a member of the viral family *Filoviridae*, was responsible for the largest recorded outbreak of filovirus disease in history, in Western Africa from 2013 to 2016. This epidemic is associated with over 28,000 suspected or confirmed cases and caused over 11,000 deaths. Genomic surveillance of viral isolates from infected patients has yielded a wealth of information on how the virus spread and evolved during the epidemic. The index case is thought to be a 4-year-old boy in Guinea, who may have been exposed to EBOV as a result of contact with an infected bat. The virus then spread to neighboring Liberia and Sierra Leone in 2014 exclusively through human-to-human transmission (1–4). The EBOV strain responsible for this outbreak was named Makona (5) and is genetically distinct from the Mayinga isolate from the first recorded EBOV outbreak (in the Democratic Republic of Congo in 1976).

Three missense mutations in the EBOV Makona genome sequence, (i) an R-to-C mutation at position 111 (R111C) in the nucleoprotein (NP), (ii) D759G in the polymerase (L), and (iii) A82V in the viral glycoprotein (GP) [GP(A82V)], arose early in the epidemic and came to dominate the viral isolates collected during the last half of 2014 and onward (6). The A82V mutation in particular was proposed to be an adaptation arising during the prolonged chain of human-to-human transmission. This is supported by the observed increase in infection of primate cells and the corollary decrease in infection of bat cell lines for viruses carrying GP(A82V) (7, 8). Although this observation has not been recapitulated in animal models of infection, including mice and rhesus macaques (9), there is mechanistic evidence that the A82V mutation impacts viral entry (10). The entry of EBOV depends on several host factors, including the obligate intracellular filovirus receptor Niemann-Pick C1 (NPC1) (11, 12) as well as cysteine cathepsin proteases that cleave GP to reveal the receptor-binding site (RBS) (13, 14). It remains unknown if the A82V mutation promotes more efficient usage of any of these known host factors or if it allows the virus to coopt novel host factors in order to mediate entry.

Another polymorphism in EBOV GP, at position 544, has also been shown to influence viral entry in tissue culture, with 544I conferring enhanced infectivity relative to 544T (15–18). Because Makona isolates have 544T, while Mayinga isolates have 544I, we postulated that the A82V mutation in Makona arose, at least in part, as an infection-promoting response to the presence of 544T. Additional differences between Mayinga GP [GP(Mayinga)] and Makona GP, such as A503V and a number of substitutions in the mucin domain, have previously been ruled out as explanations for the increased infectivity of Makona isolates bearing 82V (10).

Here, we set out to investigate the interplay between polymorphisms at positions 82 and 544 in the background of GP(Makona) and GP(Mayinga). We found that both 82V and 544I mutations accelerated the kinetics of viral membrane fusion in an additive manner. Furthermore, 82V variants were more susceptible to proteolysis by the endosomal cysteine protease cathepsin L (CatL) and displayed a striking alteration in the pattern of GP proteolytic cleavage, which correlated with enhanced viral entry under cathepsin-limited conditions. Taken together, our findings reveal a potential mechanism by which the A82V mutation enhances EBOV GP-dependent viral entry and infection.

## RESULTS

### GP variants recapitulate previously reported infection and receptor binding phenotypes.

We generated four GP variants, Makona 82A/544T, Makona 82V/544T, Mayinga 82A/544I, and Mayinga 82V/544I [here referred to as GP(Mak 82A/544T), GP(Mak 82V/544T), GP(May 82A/544I), and GP(May 82V/544I), respectively, in order to highlight the residues investigated], as vesicular stomatitis virus (VSV) pseudotypes (VSV-GP). Since none of the mutations of interest are in the GP mucin domain, and the mucin domain is dispensable for infection in cell culture (14, 19), all pseudotypes generated have a deletion of amino acid residues 309 to 489 in GP.

**FIG 1:**
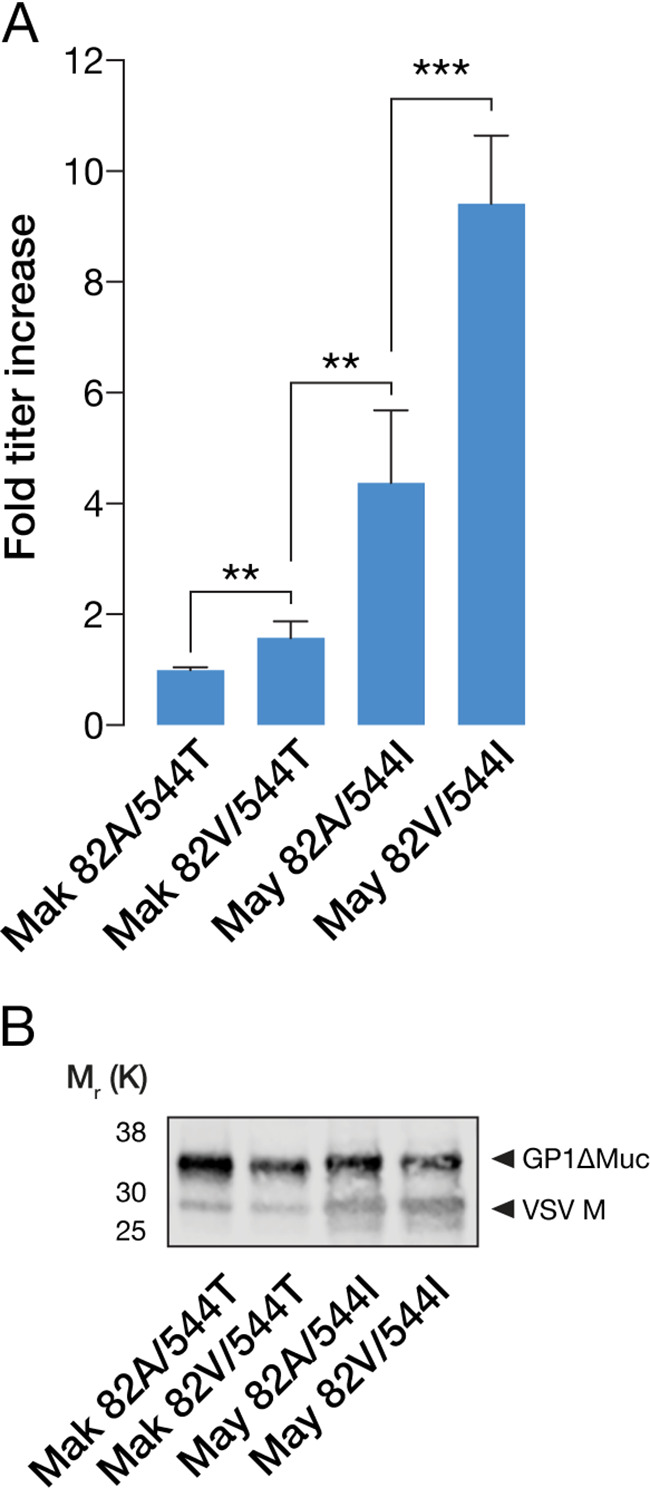
(A) Mean fold increases in viral titers (±SD) of GP variants compared to GP(Mak 82A/544T). 82V, 544I, or both in tandem significantly increase titers (**, *P* < 0.002; ***, *P* < 0.001 [by unpaired two-tailed *t* tests]) (*n* = 6, from three independent experiments). (B) Representative Western blot of VSV M and EBOV GP indicating similar levels of GP incorporation into VSV for all four GP variants.

In order to validate these pseudotyped viruses, we first measured their infectivity in Vero cells. The A82V and T544I mutations, both alone and together, promoted higher levels of infectivity (Fig. 1A), as previously reported (15–18). The differences in infectivity did not stem from differences in GP density on the viral surface, as all four variants demonstrated comparable levels of GP incorporation into particles (Fig. 1B).

**FIG 2:**
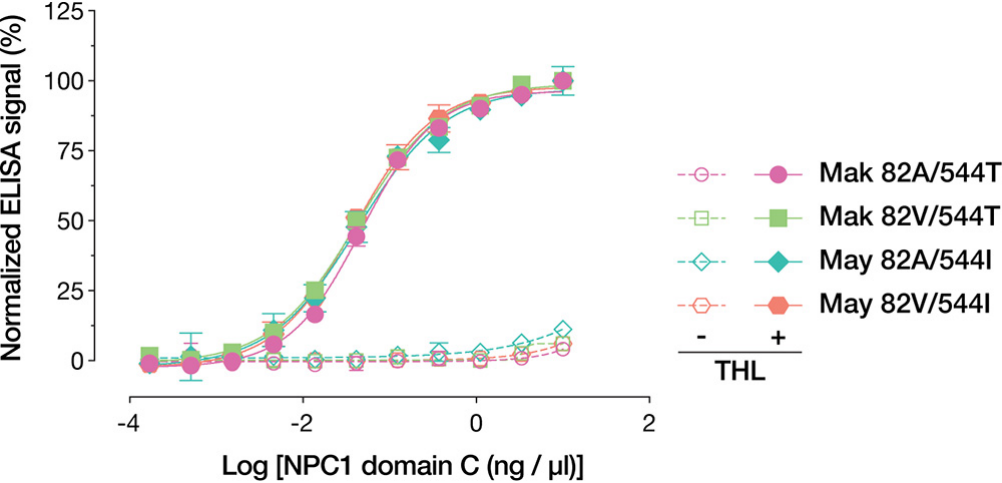
Normalized amounts of VSV-GP variants, in the native or THL-cleaved state, were captured onto ELISA plates using KZ52. Once cleaved, all four GP variants are able to bind NPC1 domain C at equal levels. Means (±SD) of results from two independent experiments with three technical replicates are shown (*n* = 6).

The various infectivity phenotypes of the mutants also could not be readily explained by differences in receptor interaction. Due to the proximity of position 82 to the RBS of GP, it has been speculated that the A82V mutation could promote binding to the critical endo/lysosomal filovirus receptor Niemann-Pick C1 (NPC1) (7, 11, 12, 20). Following pretreatment of the VSV-GPs with thermolysin (THL), which mimics the activity of cathepsin B (CatB) (14), we used an enzyme-linked immunosorbent assay (ELISA) to measure NPC1 binding to cleaved GP (GP_CL_) (21). As reported previously (10), the A82V mutation did not affect GP_CL_-NPC1 binding (Fig. 2).

**FIG 3:**
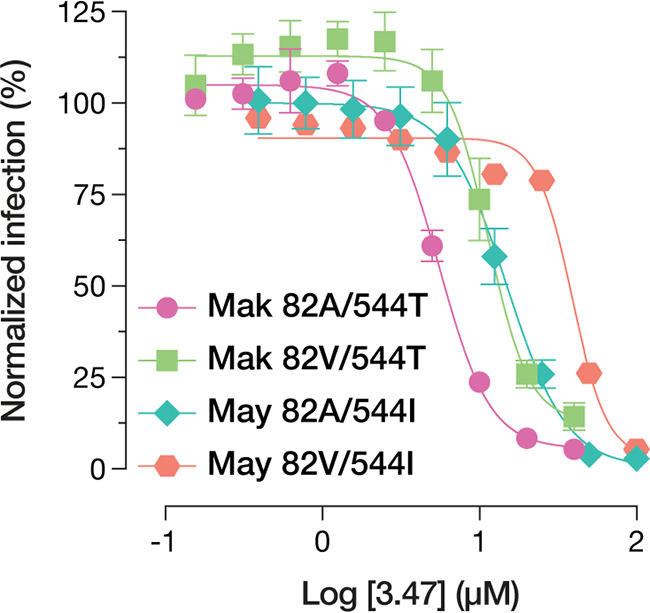
Normalized infectivity levels at various concentrations of the viral inhibitor 3.47. Means (±SD) of results from two independent experiments with three technical replicates are shown (*n* = 6). At 3.47 concentrations of >2.5 and >12.5 μM, respectively, Makona and Mayinga VSV-GP variants bearing 82V are significantly more infectious than their 82A counterparts (*P* < 0.001 [by multiple *t* tests with the Holm-Šídák correction for multiple testing]).

One of the most striking and widely reported phenotypes associated with the A82V mutation is increased resistance to the small-molecule viral inhibitor 3.47, which targets the GP-NPC1 interaction (12, 22, 23). We observed that the polymorphisms at positions 82 and 544 made independent and additive contributions to viral sensitivity to 3.47. Specifically, A82V and T544I were each associated with increased 3.47 resistance, with the 82A/544T and 82V/544I genotypes conferring low and high levels of resistance, respectively, and the mixed 82V/544T and 82A/544I genotypes conferring intermediate levels of resistance (Fig. 3). These results are largely in line with previously reported data (10) and indicate that both A82V and T544I promote viral resistance to 3.47 in a manner likely unrelated to the GP-NPC1 interaction.

### The GP-destabilizing effect of the A82V mutation is unmasked by proteolysis.

**FIG 4:**
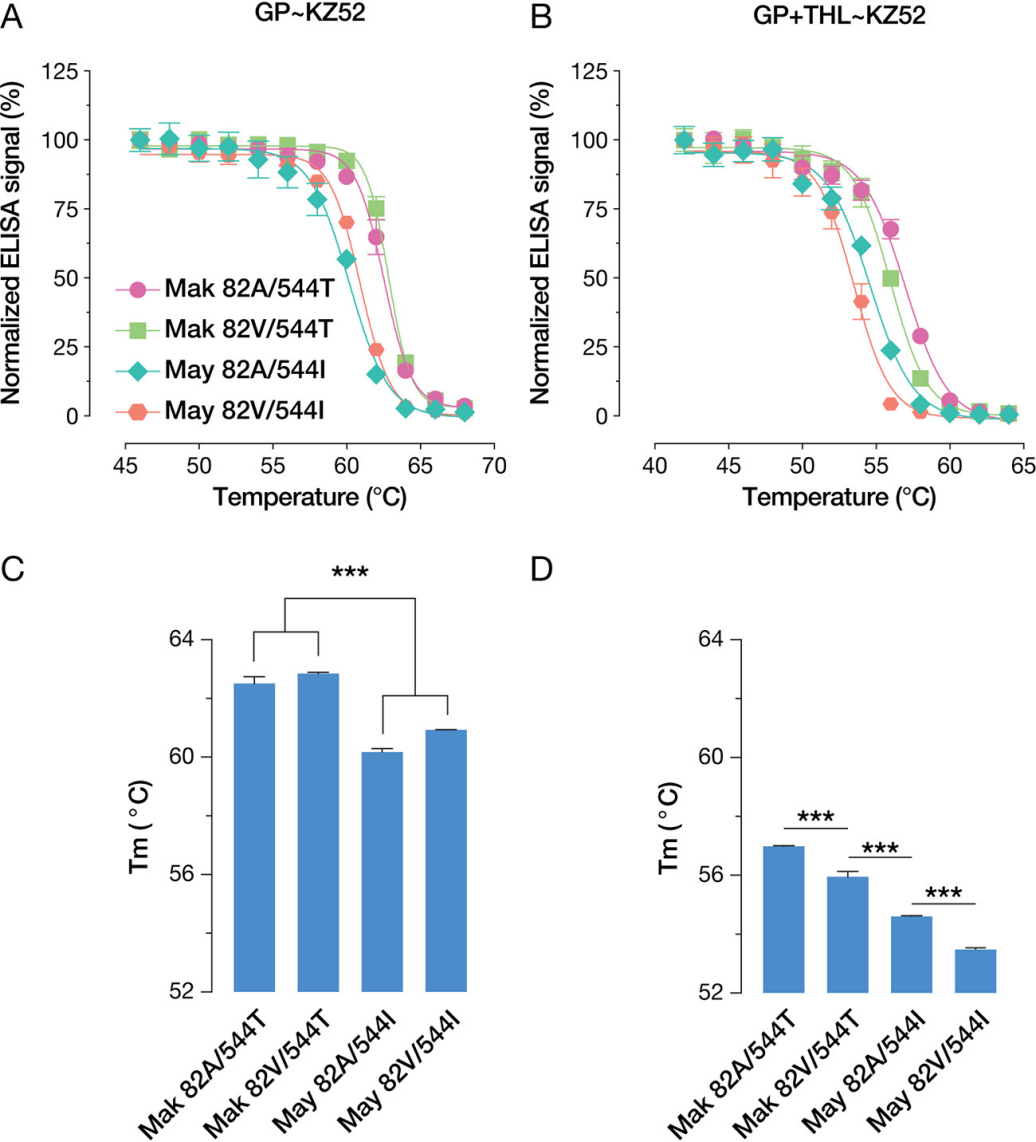
(A) Normalized ELISA signal of KZ52 binding to native VSV-GP variants following heating. Means (±SD) of results from two independent experiments with three technical replicates (*n* = 6) are shown. (B) Normalized ELISA signal of KZ52 binding to THL-cleaved VSV-GP variants following heating. Means (±SD) of results from two independent experiments with three technical replicates (*n* = 6) are shown. (C) *T_m_* values for native VSV-GP variants, derived by nonlinear regression analysis of ELISA signals. Statistical significance was determined by one-way ANOVA with Tukey correction for multiple testing (*, *P* < 0.033; **, *P* < 0.002; ***, *P* < 0.001) (D) *T_m_* values for THL-cleaved VSV-GP variants, derived by nonlinear regression analysis of ELISA signals. Statistical significance was determined by one-way ANOVA with Tukey correction for multiple testing (*, *P* < 0.033; **, *P* < 0.002; ***, *P* < 0.001).

Single point mutations have previously been shown to have dramatic effects on GP stability, with associated impacts on viral infectivity (24). A previous report implicated the reduced stability of the A82V mutant as the basis for its increased infectivity *in vitro* and *in vivo* (10). Likewise, proteolysis has a destabilizing effect on GP that is thought to promote fusion activation (24–26). Accordingly, we set out to investigate the stability of GP mutants in both uncleaved and precleaved states using an ELISA that measures the thermostability of an epitope spanning GP1 and GP2 (26). By measuring the half-maximal melting temperature (*T_m_*) of GPs, we found that uncleaved GPs bearing 544I were significantly less stable than their 544T counterparts (Fig. 4A and C). Upon THL cleavage, all GPs were destabilized, with *T_m_* values decreasing 5°C to 6°C compared to their uncleaved counterparts. Furthermore, THL cleavage unmasked more nuanced differences in stability, with GPs bearing 82V being significantly destabilized compared to the 82A variants (Fig. 4B and D). Thus, the polymorphisms at both positions 82 and 544 appear to regulate GP stability but may do so at different steps in entry: either upstream (position 544) or downstream (positions 82) of the proteolytic cleavage mimicked by THL.

### A82V and T544I increase the rate of GP fusion activation but not its probability.

**FIG 5:**
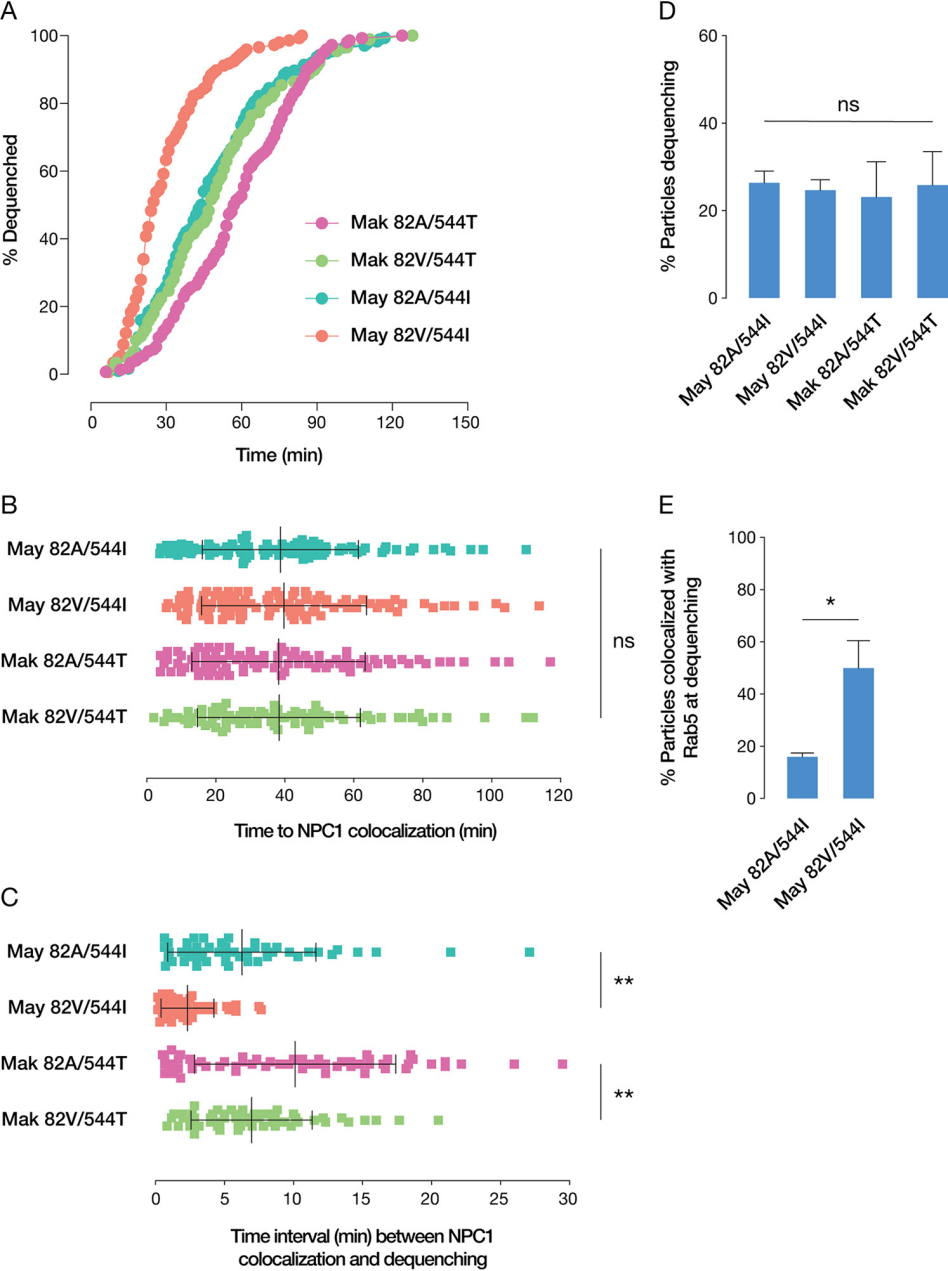
(A) Kinetics of viral fusion triggering. (B) Time of viral colocalization with NPC1. Data represent the means and SD from three independent experiments. ns, not significant. (C) Time interval between viral colocalization with NPC1 and the onset of lipid mixing. Data represent the means and SD from three independent experiments (**, *P* < 0.01 [by one-way ANOVA with a *post hoc* Tukey test]). (D) Total percentage of virions undergoing GP-mediated lipid mixing. Data represent the means and SD from three independent experiments. (E) Total percentage of virions colocalized with Rab5 at the time of lipid mixing (*, *P* < 0.05 [by Student’s *t* test]). Data represent the means and SD from three independent experiments.

In order to investigate the impact of these GP mutations on fusogenic activation, we used a fluorescence dequenching assay to observe viral fusion triggering within live cells on a single-particle basis (27, 28). Membranes of purified viruses were labeled with self-quenching concentrations of the lipophilic dye 1,1′-dioctadecyl-3,3,3′,3′-tetramethylindodicarbocyanine (DiD). Mixing of lipids between the host and viral membranes as a result of hemifusion or full fusion enables the dequenching of the dye and a sharp increase in fluorescence intensity. We observed that the polymorphisms at positions 82 and 544 made independent and additive contributions to the kinetics of fluorescence dequenching (Fig. 5A). Specifically, VSV-GP(May 82V/544I) exhibited the fastest kinetics of lipid mixing, with a *t*_1/2_ (time needed for 50% of particles to dequench) of 24 min, whereas VSV-GP(Mak 82A/544T) exhibited the slowest (*t*_1/2_ = 57 min). Viruses with the mixed GP genotypes exhibited intermediate kinetics of lipid mixing (*t*_1/2_ = 44 and 47 min for 82A/544I and 82V/544T, respectively). Interestingly, the lipid mixing kinetics of these viruses in cells were concordant with their respective susceptibilities to inhibition by 3.47 (Fig. 3).

However, despite these differences in the kinetics of GP-dependent lipid mixing in cells, we observed no difference with respect to the total percentages of cell-associated particles eventually inducing lipid mixing (Fig. 5D). Since this assay is unable to differentiate hemifusion from the formation of fusion pores and viral content release into the cytoplasm, this suggests that the differences in infectivity observed for the position 82 and 544 mutants arise at a very late step in fusion activation that we were unable to resolve other than as a change in the kinetics of fusion activation. It is also unknown how the timing of fusion impacts the probability of establishing productive infection, adding a layer of complexity to inferences about the relationship between lipid mixing and infectivity.

We also observed no significant differences among the mutants in terms of the mean time of colocalization with NPC1, demonstrating that internalization and trafficking are unaltered (Fig. 5B). However, once delivered to NPC1-positive compartments, VSV-GP(May 82V/544I) exhibited a shorter mean lag to lipid mixing (2.3 min) than GP(May 82A/544I) (6.3 min) (Fig. 5C). The lag increased slightly with GP(Mak 82V/544T) (6.9 min) and to a greater extent with GP(Mak 82A/544T) (10.1 min).

Because the dramatically accelerated fusion kinetics of GP(May 82V/544I) indicated that its threshold for triggering may be significantly reduced, we examined lipid mixing in Rab5-positive compartments (Fig. 5E). The percentage of virions undergoing dequenching in intermediate endosomes, as differentiated from early endosomes by the presence of NPC1 at the time of fusion, was significantly higher with this mutant than with GP(May 82A/544I), demonstrating that more particles can be triggered to fuse in less-mature endocytic vesicles.

### 82V variants have greater relative infectivity in CatL-deficient cells.

**FIG 6:**
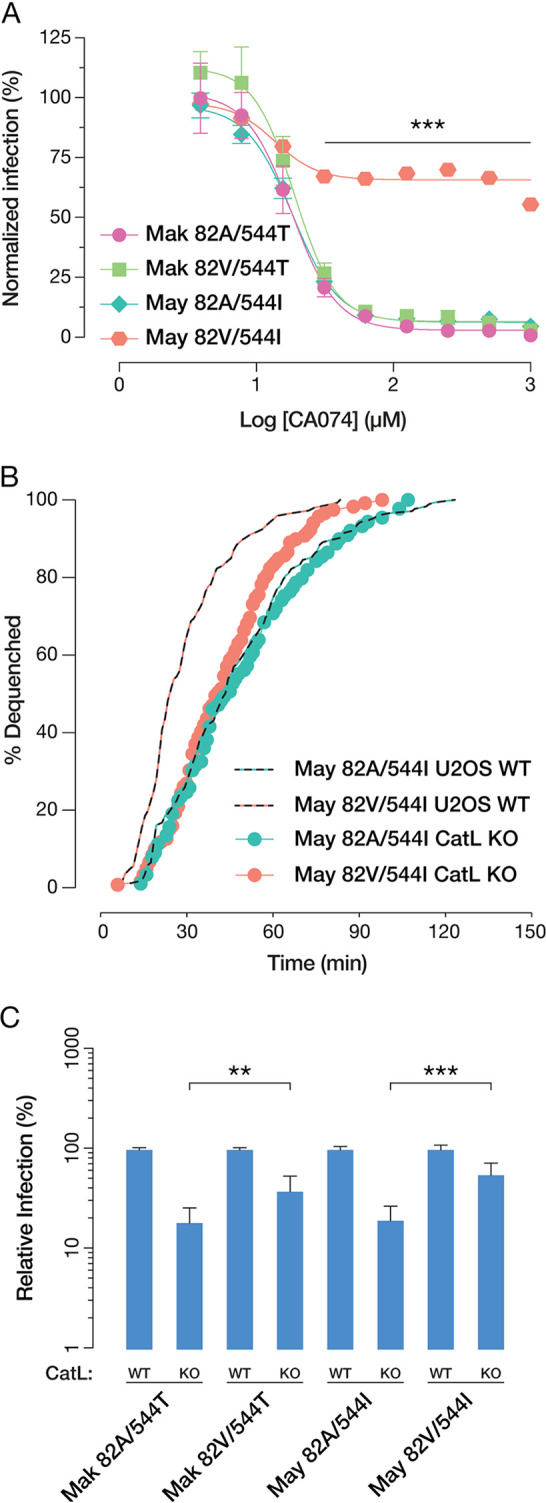
(A) Normalized infectivity levels at various concentrations of the CatB inhibitor CA074. Means (±SD) of results from two independent experiments with three technical replicates (*n* = 6) are shown. At concentrations of CA074 of >15 μM, VSV-GP(May 82V/544I) is significantly more infectious than all other GP variants (*P* < 0.001 [by multiple *t* tests with the Holm-Šídák correction for multiple testing]). (B) Kinetics of viral fusion triggering for VSV-GP(May 82V/544I) and VSV-GP(May 82A/544I) in U2OS CatL KO cells. (C) WT and CatL KO U2OS cells were infected with VSV-GP variants, and infectivity was scored by automatic counting of GFP-positive cells at 14 h postinfection. Groups were compared by one-way ANOVA with Šídák correction for multiple testing in order to determine statistical significance (*, *P* < 0.033; **, *P* < 0.002; ***, *P* < 0.001). On CatL KO cells, viruses carrying 82V variants have higher relative infectivity than those carrying 82A. Means (±SD) of results from nine trials from three independent experiments with three technical replicates are shown.

To account for the differing fusion kinetics observed among the GP variants, we next examined GP proteolytic susceptibility *in vitro*. Previous findings indicate that both the A82V and T544I mutations influence the CatB dependence of viral entry. While T544I alone does not promote resistance to the CatB inhibitor CA074, the residue at position 544 influences the overall dependence on CatB (10, 18). Here, we found that only the 82V/544I variant was resistant to CA074 (Fig. 6A), largely consistent with Wang and coworkers’ observations. Because CatL can support EBOV GP-dependent entry (13, 14, 29), we next sought to investigate the effect of the position 82/544 polymorphisms on viral entry under CatL-limited conditions. To overcome the challenges associated with selective inhibition of CatL in cells with irreversible activity-based inhibitors, we previously generated U2OS *CatL* knockout (KO) cells through CRISPR/Cas9 genome engineering (23). We then tested the kinetics of lipid mixing of the GP Mayinga variants that displayed the greatest difference on wild-type (WT) U2OS cells. To our surprise, we found that knocking out CatL drastically slowed the fusion kinetics of VSV-GP(May 82V/544I) but had a comparatively small effect on VSV-GP(May 82A/544I) (Fig. 6B). Both 82V variants afforded higher levels of infectivity than their 82A counterparts in CatL KO cells even after adjusting for their relative infectivities in the control cells (Fig. 6C). Taken together, our results suggest that A82V and T544I are required to bypass the entry requirement for CatB and that A82V drives faster fusion kinetics through a CatL-dependent mechanism. Furthermore, the relatively higher levels of infectivity of 82V variants in *CatL* KO cells indicate that they are also able to utilize non-CatL cathepsins more efficiently in order to drive viral entry.

### A82V mutations allow GP to be processed to a novel cleavage product by CatL.

**FIG 7:**
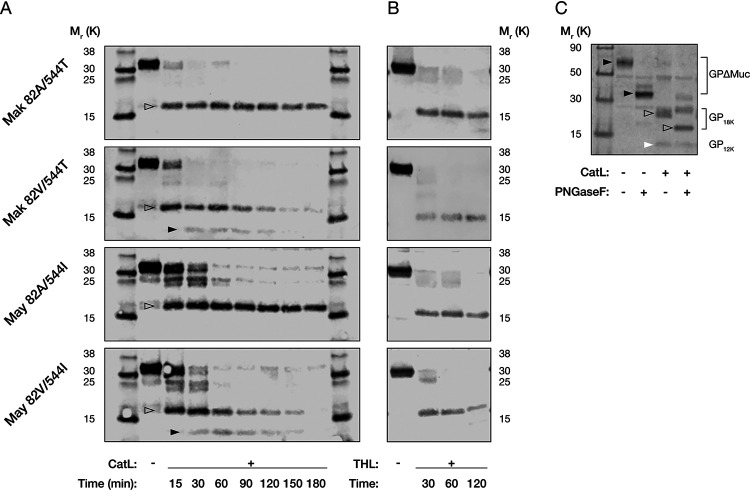
(A) Representative Western blots of GP1 CatL cleavage products analyzed by SDS-PAGE following PNGase F treatment. With increased incubation times (0 to 3 h), GP variants bearing 82V were converted to two distinct products of approximately 18K and 12K, respectively. (B) Representative Western blots of GP1 THL cleavage products analyzed by SDS-PAGE following PNGase F treatment. No overt differences in proteolysis were observed between the GP variants. (C) Representative Western blot of CatL-treated and native VSV-GP(May 82V/544I), with or without PNGase F treatment, showing the glycosylation-dependent migration of GP (black arrows), GP_18K_ (open arrows), and GP_12K_ (white arrow).

To explore the molecular basis of enhanced viral entry in CatL-deficient cells afforded by the A82V mutation, we subjected VSVs bearing all four GP(82/544) variants to *in vitro* proteolytic cleavage with CatL or THL (to mimic CatB activity) and analyzed the deglycosylated cleavage products by SDS-PAGE (Fig. 7A). With CatL treatment, we observed a rapid conversion to an 18,000-molecular-weight (18K) species (GP1_18K_) for all four GP variants. GPs with 82A were resistant to further proteolysis, as evidenced by the persistence of GP1_18K_ for up to 3 h. GPs with 82V, in contrast, were converted to a novel GP1 cleavage product of ∼12K in size (GP1_12K_). Both GP1_18K_ and GP1_12K_ were degraded upon prolonged CatL treatment. Treatment with THL led only to the formation of the expected 17K product (GP1_17K_) for all four GP variants. No overt differences in susceptibility to THL were observed in 1 h of THL treatment. Therefore, the presence of 82V appears to induce a specific change in GP sensitivity to CatL rather than a generalized change in its sensitivity to proteolysis (Fig. 7B).

**FIG 8:**
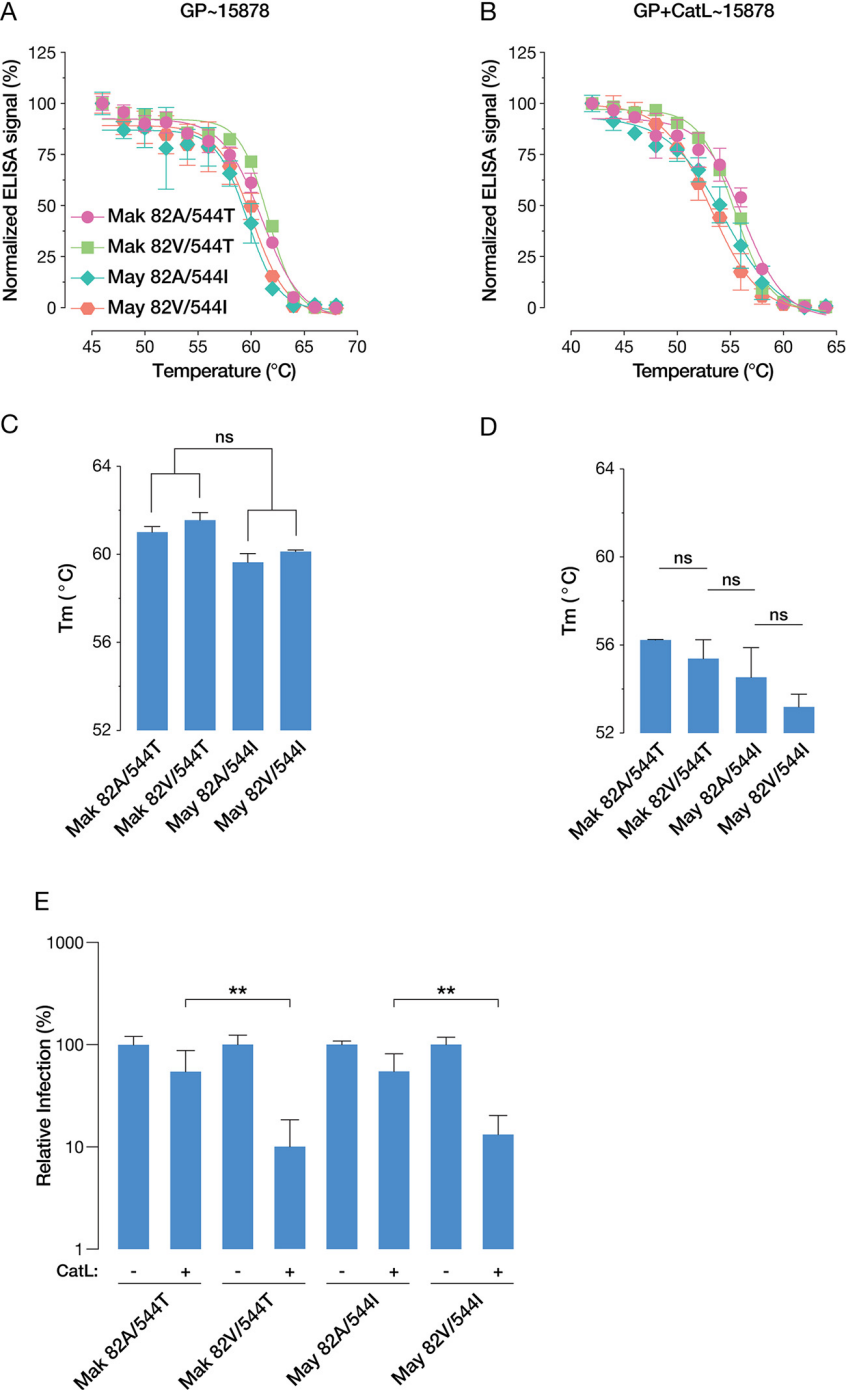
(A and B) Normalized ELISA signals of ADI-15878 binding to native (A) and CatL-treated (B) VSV-GP variants following heating. Means (±SD) of results from two independent experiments with three technical replicates (*n* = 6) are shown. (C and D) *T_m_* values for native (C) and CatL-treated (D) VSV-GP variants, derived by nonlinear regression analysis of ELISA signals. Statistical significance was determined by one-way ANOVA with Tukey correction for multiple testing (ns, not significant [*P* > 0.05]). (E) Relative infectivity of native and CatL-treated VSV-GP variants. Statistical significance was determined by one-way ANOVA with Šídák correction for multiple testing. Means (±SD) of results from two independent experiments with three technical replicates (*n* = 6) are shown.

Given that proteolysis by THL destabilized GPs bearing 82V, we investigated if proteolysis by CatL to this novel GP cleavage product represents an additionally destabilizing step. Accordingly, we exposed VSV-GP variants to CatL for 1 h, at which point a maximal ratio of GP_12K_ to GP_18K_ was achieved, before measuring GP thermostability. Since CatL-cleaved GP is not recognized by KZ52, we made use of a monoclonal antibody (mAb) (ADI-15878) targeting a partially overlapping epitope in order to measure conformational intactness following heating (26, 30). We observed a pattern of melting temperatures that is strikingly similar to that of THL-treated GP, although the differences between GP variants were not statistically significant (Fig. 8A to D). Thus, the appearance of the GP_12K_ cleavage product does not seem to be accompanied by any detectable additional destabilization of GP.

We also measured the relative infectivity of VSV-GP variants following a 1-h incubation with CatL. While all variants displayed a decreased level of relative infectivity, the reduction in viral titers of variants bearing 82V was significantly larger than that of variants bearing 82A (Fig. 8E), indicating that the generation of the GP_12K_ species by precleavage with CatL is inactivating. We conclude that, unlike GP_18K_ and GP_17K_, GP_12K_ does not represent a more fully primed prefusion conformer of GP and speculate that its generation must be coupled to cell entry in order to drive enhanced increased infectivity.

Because GP1_18K_ appears to chase into GP1_12K_, the latter must be derived by the cleavage of the former. Accordingly, we next sought to establish which GP1 sequences are lost upon GP1_18K_→GP1_12K_ cleavage. Previous work indicates that CatL-cleaved WT Mayinga GP1_18K_ comprises GP1 residues 33 to 190 (31). Making use of the N-linked glycan at position 40 of GP1_18K_, the sole remaining glycan in this product (32), we analyzed the migration patterns of the VSV-GP(May 82V/544I) cleavage products prior to or following deglycosylation with protein-*N*-glycosidase F (PNGase F). For intact GP1ΔMuc and GP1_18K_, we observed a shift in the electrophoretic mobility of these products upon PNGase F treatment, whereas GP1_12K_ was unaffected (Fig. 7C). Thus, N-terminal residues encompassing N40 are removed during GP1_18K_→GP_12K_ cleavage. Moreover, GP_12K_ was recognized by an antiserum raised against a peptide comprising GP1 residues 83 to 97 (13) (Fig. 7A), indicating that it still contains this sequence. We propose, therefore, that the new N-terminus generated by GP1_18K_→GP_12K_ cleavage lies between GP1 residues 41 and 83. Whether GP1_18K_ also undergoes C-terminal cleavage remains unknown at present.

## DISCUSSION

The 2013–2016 EBOV epidemic in West Africa led to an unprecedented number of infections and deaths but has enabled the first opportunity to examine how the virus evolves during prolonged human-to-human transmission. Viral sequences sampled during this epidemic revealed several nonsynonymous mutations compared to the EBOV Mayinga reference strain isolated in 1976 following limited human-to-human transmission (1–4). Two of these polymorphisms in GP, at positions 82 and 544, were previously shown to have opposing impacts on viral infectivity (15, 16). Here, we show that these mutations affect distinct steps in EBOV entry, which points toward a novel mechanistic basis for the increased infectivity of the A82V mutation.

EBOV entry into cells depends on a number of host factors, including the cysteine proteases CatB and CatL that act on GP to reveal the binding site (13, 14) for the universal filovirus receptor NPC1 (11, 12). Evidence suggests that these factors, while critical for entry, are insufficient to elicit GP membrane fusion (20, 33, 34) and that an additional host factor, acting as a fusion trigger, must exist.

Although viral fusion glycoproteins can be primed and triggered by many different factors, thermostability serves as a good general proxy for their capacity to undergo fusogenic conformational changes (24, 35, 36). We found that GPs bearing 544I were less thermostable than GPs bearing 544T, regardless of the residue at position 82, and that THL-cleaved GP exhibited incremental destabilization, with both 82V and 544I contributing to reduced stability versus their 82A and 544T counterparts. Similar results were observed for CatL-cleaved GPs, suggesting that the differences observed between the GP variants are not protease specific but rather reflect general changes in stability. We propose that polymorphisms at position 544 primarily influence global GP stability, which may have ramifications for initial proteolytic cleavages in GP or even for fusogenic rearrangement. Due to its location in the GP2 internal fusion loop, residue 544 has been speculated to alter fusogenicity and/or bilayer insertion (10, 17, 18, 37). In GP peptides at low pH, 544I forms part of a “hydrophobic fist” together with 529L and 535F, which is proposed to be necessary for penetration of the host membrane (38). Although this study did not examine the effect of polymorphisms at position 544, we found no gross defect in fusion triggering or hemifusion in cells, only a significant delay in fusion kinetics between mutants bearing T or I at this position.

Although 544I has been demonstrated to increase infectivity in cell lines derived from different hosts, the promotion of infection by 82V occurs selectively (37), suggesting that its basis for enhanced infectivity is host species dependent. Some evidence for 82V-induced instability lies in the mutant’s 3.47 resistance and decreased *T_m_* values, but we argue that this is secondary to 82V’s role in facilitating GP cleavage by CatL. Cell type-dependent differences in CatL abundance or activity may explain the selectively heightened infectivity of 82V better than differences in NPC1 interaction.

The relative instability of GP bearing 82V has been cited as the primary source of its enhanced infectivity (10). Interestingly, we saw that fusion triggering occurred more rapidly for GPs bearing 82V, 544I, or a combination thereof, as measured by overall lipid mixing rates and time to fusion following delivery to NPC1-positive compartments. Although our live assays were unable to monitor the infection success of any given membrane fusion event, earlier triggering of viruses is generally associated with increased infectivity (39, 40). In addition to driving more rapid viral entry, the higher rates of fusion may enhance the probability of successful viral escape into the cytoplasm by facilitating evasion of the harsh conditions in the lysosome and/or lysosome-targeted host antiviral factors such as the late endosomal/lysosomal interferon-induced transmembrane proteins (IFITMs) (28, 41). We also found that the high rate of lipid mixing of 82V variants is mediated by a CatL-dependent activity, as the kinetics of lipid mixing by VSV-GP(May 82V/544I) and VSV-GP(May 82A/544I) were largely the same in cells lacking CatL.

These observations are in contrast to the *in vitro* proteolytic cleavage of GP by CatL, which is promoted specifically by the presence of 82V but not 544I. GPs bearing 82V were both more labile and, intriguingly, processed to a novel GP1 cleavage product of approximately 12K in size (GP1_12K_). Our approximate mapping of this newly identified novel cleavage product indicates that the GP1_18K_ product usually obtained with WT GP undergoes further N-terminal cleavage by CatL to remove as many as 50 additional amino acid residues. This altered pattern of proteolytic cleavage was not observed following treatment with THL, but it may extend beyond CatL in cells since a relative increase in infectivity was observed in U2OS cells lacking CatL. If cleaved by CatL in order to enrich for GP_12K_, 82V variants instead display decreased infectivity, suggesting that this product could represent a pretriggered form of GP. The temporal and spatial coordination of the generation of this form of GP may be required to drive enhanced viral entry by GP variants bearing A82V.

Our findings suggest that residue 544 acts as a regulator of infectivity in cell culture by modulating overall GP stability and fusogenicity, whereas residue 82 more specifically influences proteolytic cleavage of GP by host endosomal cysteine cathepsins. This is reflected in increased relative infectivity under conditions where cysteine cathepsin activity is depleted, either genetically or by chemical inhibition, as well as in increased proteolysis *in vitro* with the appearance of a novel cleavage product. Combining these effects affords an increase in the kinetics of fusion, explaining the previously observed increase in infectivity of the GP(82V/544I) double mutant.

We recently showed that a mutation near the N terminus of GP1, R64A, inhibited viral membrane fusion and entry in a manner reversible by second-site mutations near the GP1 N terminus (24). These second-site reversions were predicted to destabilize GP and enhance the exposure of GP1 N-terminal sequences to proteolysis. Our current findings argue that A82V accelerates viral membrane fusion and increases viral entry through a related mechanism: enhancing proteolytic cleavage at N-terminal sequences in GP1. Although more work is needed, both sets of observations are concordant with a hypothesis in which conformational changes at the GP1 base, coupled to NPC1 recognition by the receptor-binding site in the GP1 head, expose the base to further proteolytic cleavages that drive fusogenic rearrangement. We speculate that these GP1 N-terminal cleavages are normally mediated by the sequential activity of endosomal cysteine aminopeptidases such as CatC or CatH but can be more promiscuously carried out by cysteine endoproteases like CatL in the context of the A82V mutation, thereby contributing to the observed enhancements in viral entry and infection.

## MATERIALS AND METHODS

### Cells, viruses, and infection conditions.

All infection experiments were carried out in Vero cells cultured in Dulbecco’s modified Eagle medium (DMEM) (Life Technologies, Carlsbad, CA) supplemented with 2% fetal bovine serum (FBS) (Atlanta Biologicals, Flowery Branch, GA), 1% penicillin-streptomycin (Life Technologies, Carlsbad, CA), and 1% GlutaMAX (Life Technologies, Carlsbad, CA) or in U2OS cells cultured in McCoy’s 5A modified medium (Life Technologies, Carlsbad, CA) supplemented with 10% fetal bovine serum (Atlanta Biologicals, Flowery Branch, GA), 1% penicillin-streptomycin (Life Technologies, Carlsbad, CA), and 1% GlutaMAX (Life Technologies, Carlsbad, CA). U2OS cells stably expressing monomeric NeonGreen fused to NPC1 (NPC1-mNG) were generated as previously described (27).

Pseudotyped VSVs bearing mutant EBOV GPs were generated as described previously (29, 42). Mutant Mayinga GPs were based on the EBOV/H.sapiens-tc/COD/1976/Yambuku-Mayinga isolate amino acid sequence (GenBank accession number AF086833). Mutant Makona GPs were based on EBOV/H.sapiens-wt/LBR/2015/Makona-LIBR16393 (GenBank accession number KY744596). All GPs included a deletion of the mucin domain (Δ309–489) (43).

Prior to infection experiments, confluent Vero or U2OS cells were seeded in their respective growth media described above. VSVs bearing mutant GPs were diluted in the corresponding media before addition to cells. Infected cells were then maintained at 37°C for 14 to 16 h postinfection before automatic counting of green fluorescent protein (GFP)-positive cells using a Cytation 5 cell imaging multimode reader (BioTek Instruments, Winooski, VT) and using the onboard software for calculations of the percentage of infected cells.

### NPC1 domain C ELISA.

Plates were coated with GP-specific mAb KZ52 (44) diluted to 2 μg/ml in phosphate-buffered saline (PBS). VSV-GP mutants were normalized for GP content and then incubated at 37°C for 1 h in the presence of 250 μg/ml thermolysin (Sigma-Aldrich, St. Louis, MO), before the addition of 10 mM phosphoramidon to stop the reaction. ELISA plates were blocked using PBS supplemented with 3% bovine serum albumin (PBSA) (Thermo Fisher Scientific, Waltham, MA). THL-treated virus was added to blocked KZ52-coated plates and allowed to adsorb at 37°C for 1 h. Following washing with 3% PBSA, a dilution series of FLAG-tagged NPC1 domain C (45) was added and allowed to bind for 1 h at 37°C. Bound domain C was then detected using a horseradish peroxidase (HRP)-conjugated anti-FLAG antibody (Ab) (Sigma-Aldrich, St. Louis, MO) and the Ultra-TMB substrate (Thermo Fisher Scientific, Waltham, MA). Fifty percent effective concentration (EC_50_) values were calculated using nonlinear regression of data from two independent experiments with three technical replicates each.

### CA074 and 3.47 inhibitor experiments.

The viral inhibitors 3.47 (Microbiotix, Worcester, MA) and CA074 (Sigma-Aldrich, St. Louis, MO) were reconstituted in dimethyl sulfoxide (DMSO). Twofold dilution series starting at 100 μM and 1 mM, respectively, were then made in complete DMEM (Life Technologies, Grand Island, NY) supplemented with 2% fetal bovine serum (Atlanta Biologicals, Flowery Branch, GA), 1% penicillin-streptomycin (Life Technologies, Carlsbad, CA), and 1% GlutaMAX (Life Technologies, Carlsbad, CA). Diluted inhibitors were then added to confluent Vero cells, and uptake was allowed to proceed through 4 h of incubation at 37°C. VSV-GP mutants were then diluted 1:1,000 in complete DMEM and added to inhibitor-treated cells. At 14 to 16 h postinfection, cells were fixed in 4% paraformaldehyde (PFA) and stained with Hoechst nuclear stain before GFP-positive cells were counted using a Cytation 5 cell imaging multimode reader (BioTek Instruments, Winooski, VT) with the onboard software for calculations of the percentage of infected cells.

### *In vitro* proteolysis.

VSV-GP mutants, normalized for GP content and corresponding to between 2 × 10^5^ and 2 × 10^6^ IU, were incubated with activated human recombinant CatL (R&D Systems, Minneapolis, MN) at 2 ng/μl or THL (Sigma-Aldrich, St. Louis, MO) at 250 μg/ml. Cleavage by CatL was allowed to proceed at 37°C for 15, 30, 60, 90, 120, 150, or 180 min before the reaction was stopped by the addition of 0.1 mM E-64. Cleavage by THL was allowed to proceed at 37°C for 30, 60, or 120 min before the reaction was stopped by the addition of 10 mM phosphoramidon. Cleavage products were then deglycosylated by treatment with 250 U PNGase F (New England BioLabs, Ipswich, MA) for 16 h at 37°C. Parallel samples of VSV-GP(May 82V/544I) treated with CatL for 60 min were also left without deglycosylation for the analysis of the presence of the glycan at N40.

### SDS-PAGE and Western blotting.

Deglycosylated and native samples containing GP cleavage products were analyzed by SDS-PAGE using 10% Tricine protein gels (Thermo Fisher Scientific, Waltham, MA). Samples were transferred onto 0.2-μm-pore-size Protran nitrocellulose membranes (Sigma-Aldrich, St. Louis, MO) followed by Western blotting for GP1 using a rabbit polyclonal serum recognizing a peptide (TKRWGFRSGVPPKVV) overlapping the receptor-binding site. IRDye 680LT goat anti-rabbit IgG 680 secondary Ab (Li-Cor, Lincoln, NE) was used at a dilution of 1:10,000, and the final blot was then imaged using a Li-Cor Fc fluorescence imager.

### GP thermostability assay.

A GP thermostability assay was conducted as previously described (24). VSVs bearing mutant GPs were treated with THL at 500 μg/ml for 30 min at 37°C, or mock treated, before the addition of 10 mM phosphoramidon to stop the reaction. Cleaved and uncleaved viruses were diluted in PBS and then heated at a range of temperatures spanning from 42°C to 64°C (cleaved) and 46°C to 68°C (uncleaved) for 10 min, followed by cooling to 4°C using a thermal cycler (Applied Biosystems, Foster City, CA). After cooling, virus was directly captured onto high-binding 96-well half-area ELISA plates (Corning, Corning, NY). Plates were then blocked using 3% bovine serum albumin in PBS. GP was detected using KZ52 or ADI-15878, two conformation-specific anti-EBOV GP monoclonal antibodies. Antibody bound to GP was then detected with anti-human antibody conjugated to HRP (EMD Millipore, Burlington, MA) and the Ultra-TMB substrate (Thermo Fisher, Grand Island, NY). All binding steps were carried out at 37°C for 1 h. Binding curves were generated using Prism (nonlinear regression, variable slope [four parameters]; GraphPad Software, La Jolla, CA). *T_m_* values were calculated from two independent experiments, each with three replicates.

### Virus labeling for live-cell microscopy.

Purified virus (1 mg/ml) was labeled with 50 μM 1,1′-dioctadecyl-3,3,3′,3′-tetramethylindodicarbocyanine (DiD) dye while being agitated for 1 h at 4°C. Excess dye was removed by ultracentrifugation of the virus through a 10% sucrose cushion for 2 h at 107,000 × *g* at 4°C using an SW41 rotor (Beckman Coulter). Labeled virus pellets were resuspended at a viral protein concentration of 1 mg/ml, aliquoted, and stored at −80°C until use.

### Live imaging.

Live-cell microscopy was performed as previously described (28) with an AxioObserver.Z1 wide-field epifluorescence microscope (Zeiss, Oberkochen, Germany) equipped with a 40×/1.3-numerical-aperture (NA) objective, a DAPI (4′,6-diamidino-2-phenylindole)/GFP/Texas Red/Cy5 filter set, and a heated environmental enclosure maintained at 37°C. U2OS cell monolayers were seeded onto fibronectin-coated 35-mm glass coverslip dishes (MatTek, Ashland, MA) 24 h before experiments. Cells were chilled for several minutes on ice before spinoculation of DiD-labeled virus onto monolayers at 1,500 × *g* at 6°C for 20 min. Unbound particles were removed by five washes with cold PBS, and 500 μl cold imaging buffer (140 mM NaCl, 2.5 mM KCl, 1.8 mM MgCl_2_, 20 mM HEPES, 5 mM sucrose, 2 μM Hoechst 33342, and 2% FBS) was added to cover the cells. The dish was immediately mounted on the microscope objective and focused. The coverslip dish was then flooded with 1.5 ml warm imaging buffer to mark the start of experiments (*t *= 0). Images were acquired every 10 s for the duration of the experiments using a single z-section, which encompassed nearly all cell-associated particles.

### Data analysis.

Image analysis and single-particle tracking were performed using Volocity software (PerkinElmer, Waltham, MA) as previously described (27). Image files were not manipulated, apart from minor adjustments in brightness and contrast. Viral puncta were thresholded by initial intensity and size. Puncta falling outside the range of the 0.25 to 1 μm^2^ expected of individual DiD-labeled virions were excluded from single-particle analysis. Virions were considered colocalized with NPC1 or Rab5 only if the cellular marker punctum exceeded the background signal by 30% or more and if the intracellular and viral puncta cotrafficked with a >70% overlap of signals. Mean measurements (± standard deviations [SD]) were derived from three separate experiments unless otherwise indicated.

### Statistics and reproducibility.

Experiments were performed using two independently generated viral stocks or one viral stock benchmarked to historical stocks in terms of titer and GP incorporation. All data represent results from two or three independent experiments, each with two or three replicates (*n* = 6 in all instances). Western blots are representative of experiments performed at least 3 times.

The appropriate statistical analysis was applied to each experiment. Viral titers were compared by unpaired two-tailed *t* tests. Sensitivities to 3.47 and CA074 were compared by multiple *t* tests, with Holm-Šídák correction for multiple testing. Melting temperatures and lipid mixing kinetics were compared using one-way analysis of variance (ANOVA) with Tukey correction for multiple testing. Relative infectivities in CatL KO cells, and by CatL-treated virus, were compared by one-way ANOVA with Šídák correction for multiple testing. Significance levels are indicated in each figure legend through the use of asterisks.
